# Staging and Grading in Gerodontology—An Introduction

**DOI:** 10.1111/ger.70060

**Published:** 2026-05-27

**Authors:** W. Murray Thomson, Gerry McKenna, Murali Srinivasan, Frauke Müller, Falk Schwendicke

**Affiliations:** ^1^ Faculty of Dentistry The University of Otago Dunedin New Zealand; ^2^ Centre for Public Health Queen's University Belfast Belfast UK; ^3^ Clinic of General‐, Special Care‐ and Geriatric Dentistry, Center for Dental Medicine University of Zurich Zurich Switzerland; ^4^ Department of Physiology, Faculty of Dentistry, Center of Excellence in Precision Medicine and Digital Health Chulalongkorn University Bangkok Thailand; ^5^ Unit of Gerodontology and Removable Prosthodontics, University Clinics of Dental Medicine University of Geneva Geneva Switzerland; ^6^ Department of Conservative Dentistry, Periodontology and Digital Dentistry LMU University Hospital, LMU Munich Munich Germany

**Keywords:** case complexity, frailty, hyposalivation, nutrition, older adults, oral health care, socio‐economic status, treatment planning, xerostomia

## Abstract

**Introduction:**

Gerodontology lacks a unified framework to describe case complexity and assess the multidimensional determinants shaping oral health.

**Methods:**

An international initiative developed an evidence‐based case definition for staging and grading older adults' oral health.

**Results:**

Staging includes three domains: person, oral function (clinical and subjective), and oral disease (caries, periodontal disease, mucosal conditions) level staging. Grading incorporates risk factors at the oral, systemic, and social levels, including caries and periodontal risk, prosthetic maintenance, xerostomia, multimorbidity/polypharmacy, care dependency, nutritional status, socioeconomic position, and social support.

**Conclusions:**

This framework integrates biological, functional, systemic, and social factors within a single structure and reached strong international consensus. It offers a common language for clinical care, research, education, and policy. Further work should evaluate its feasibility, validity, and responsiveness. Companion papers in this issue provide the detailed staging and grading criteria.

Defining the oral health status and care needs of older adults is complex. Ageing populations exhibit a broad spectrum of function, frailty, and dependency. Many older individuals have retained their natural teeth and complex prostheses, while others are care‐dependent and at high risk of rapid deterioration. Current diagnostic and remuneration systems do not reflect this heterogeneity, however.

The field of gerodontology operates in the overlap of clinical care, prevention, and social support. Without an agreed framework to describe case complexity, comparisons across populations or settings remain inconsistent. Existing screening instruments primarily identify treatment need or referral urgency, but few capture multidimensional determinants such as frailty, systemic disease, or the social context.

To address this gap, an international initiative was launched to develop a reproducible, evidence‐based case definition for characterising older adults' oral health. The project aimed to provide a structure for staging (current status) and grading (future risk) that could be useful across clinical, educational, research, and policy domains. Together, staging and grading should permit a multidimensional assessment of oral health and its determinants in older adults. Detailed descriptions of staging and grading (as well as the methodology involved) appear in companion manuscripts in this special issue of *Gerodontology* [1, 2].

Staging includes three hierarchical levels:

1. Person‐level staging, based on the Clinical Frailty Scale, categorising individuals into three stages of fitness or dependency;
2. Oral function‐level staging, distinguishing both clinical function (shortened dental arch and bilateral occlusal support) and subjective function (patient‐reported impacts on eating, speaking, smiling, and comfort); and
3. Oral disease‐level staging, summarising the presence and severity of dental caries, periodontal disease, and mucosal conditions.

These domains jointly describe an individual's current oral health and care complexity, serving as the entry point for case definition.

Grading evaluates oral‐level, systemic‐level, and social‐level risk factors:

- Oral‐level grading of caries risk, periodontal progression risk, and prosthetic maintenance demands;
- Systemic‐level grading of the severity of xerostomia, degree of multimorbidity/polypharmacy and care dependency, and nutritional status; and
- Social‐level grading of social status (using the MacArthur Scale or equivalent) and social support (living arrangements and assistance availability).

This new framework establishes a unified approach for describing oral health in older adults. By distinguishing between staging (current condition) and grading (future risk), the framework integrates biological, functional, systemic, and social determinants within a single structure. It could support clinical decision‐making, research stratification, educational curricula, or policy planning. The development process was international and evidence‐driven, combining systematic reviews and structured consensus methodology. All derived statements reached strong agreement, suggesting broad acceptability. Further research should evaluate its feasibility in diverse clinical contexts, along with its validity and responsiveness to change (whether through clinical intervention or natural deterioration).

The developed framework provides a comprehensive means to describe oral health in older adults, encompassing frailty, function, disease, systemic factors, and social context. It should facilitate communication among clinicians, researchers, educators, and policymakers. The accompanying papers detail the full staging and grading criteria and serve as reference resources for those adopting the framework in practice or research. The consensus group and the editors of *Gerodontology* encourage assessment, validation, and implementation of the framework.

## Author Contributions

Each author made substantial contributions to the conception and design of the work and/or the acquisition, analysis, or interpretation of data; participated in drafting the manuscript or revising it critically for important intellectual content; approved the final version to be published; and agreed to be accountable for all aspects of the work.

## Funding

The consensus meeting was supported by the Gerodontology Association.

## Ethics Statement

The authors have nothing to report.

## Consent

All authors agreed to publishing.

## Conflicts of Interest

The authors declare no conflicts of interest.

## Data Availability

No datasets were generated or analysed during the current study.
